# An experiment in education for automatic analysis

**DOI:** 10.1155/S1463924679000729

**Published:** 1979

**Authors:** D. Betteridge

**Affiliations:** Chemistry Department University College of Swansea Swansea SA2 8PP UK


### An experiment in education for automatic analysis

**D. Betteridge**

Chemistry Department, University College of Swansea, Swansea SA2 8PP, U.K.

In July 1979 a Summer School in Automatic Analysis was organised by D.G. Porter, P.B. Stockwell, both of the Laboratory of the Government Chemist, and the author, under the auspices of the Royal Institute of Chemistry/Chemical Society. Its prime objective was to provide an up-to-date account of the philosophy and practice of automatic analysis. The audience was drawn mainly from industry and the school lasted for one week. To the best of our knowledge it was the first attempt to provide a course of theory and experiment on *all* aspects of automatic analysis.

Preliminary discussions when planning the school showed that there is no consensus about what constitutes an education in automatic analysis and how it is obtained, although it is undoubtedly one of the most important issues of contemporary analytical chemistry. This article attempts to define the scope of the challenge, in the light of the experience gained in July's experiment in education.

### Organisation of the summer school

The Summer School comprised a series of lectures, tutorials, practical exercises and demonstrations of equipment. The overall content and balance is indicated in Table 1.

**Table 1. Organisation of Summer School**

| Form of teaching | Time allocated | Instructors |
|---|---|---|
| Lectures | 16x45 min = 12 hrs | H. Bartels, Discrete analysers<br>D. Betteridge, Microprocessors<br>G.K.E. Copeland, Tobacco Smoke Problem<br>D.R. Deans, GLC<br>M.B. Denton, Spectrochemical methods, Future of automation<br>J.K. Foreman, Management<br>E.H. Hansen, Flow injection analysis<br>F.L. Mitchell, Introduction to Automation, New Techniques in Clinical analysis<br>H.L. Pardue, New Electronics, Kinetics<br>D.G. Porter, Make or Buy<br>J. Ruzicka, Flow injection analysis<br>P.G. Sauders, Clinical<br>P.B. Stockwell, Computerisation or Automation? |
| Tutorials | (a) Course problem 3x45 min<br>(b) Specialist 4x45 min<br>Total-5¼ hrs | Lecturers and T. Alliston, B. Karlberg, I. Scott, J.M. Skinner, I. Telford |
| Practicals | (a) Fixed exercises 4x1½ hr<br>(b) Free choice 2x1 hr<br>+ Wed.afternoon<br>Total=(6+3)hr | Beckman, Bifok, Chem Lab C.I. Electronics, E.D.T, Mettler, Phase Separations, Pye, Spectra Physics, Technicon, Varian, Vickers, Laboratory of the Government Chemist, ICI Petrochemicals. |

### Lectures

The lecturers were briefed to be clear and to deal with the fundamentals; to teach not to present a research paper. The exception was Professor Denton who was asked to let rip as he wished, and whose lecture on the future of automation was outstanding. The rest of the lectures, albeit many dealing with the most recent developments, were presented so as to be of immediate practical value to the participants.

### Tutorials

The tutorials, or small group discussions, were of two kinds. One set was arranged by the indicated preferences of the participants, who then had the opportunity for discussing their specific problems with an expert. The other, with fixed groups and tutor, was devoted to the course problem. This was based on the experience of the Laboratory of the Government Chemist in automating the analysis of cigarettes to measure their tar, nicotine and carbon monoxide levels. There were eight groups with five students in each and they all independently worked out a solution to the problem. Then a snowballing procedure was adopted to present the best of the individual efforts for discussion. First the (primary) groups were combined to give three (secondary) groups of 15 (or more accurately 2x15 and 1x10). The primary groups had appointed a spokesman to present the group view and the secondary groups in turn nominated a spokesman to represent their group's views. Finally these three spokesmen presented the views of the combined groups to the whole school. The procedure ensured that discussion at the end was relevant, and organised. Furthermore there was considerable interest in finding out what the others had worked out. As an additional bonus, the presentations were varied, amusing and of a very high standard. It is clear that snowballing is excellent for group discussions, it allows everyone to express his views (simultaneously, but in different rooms) and ensures that the discussion en masse is neither dominated by extroverts with an axe to grind nor falls flat because people are overawed by the occasion.

### Practicals

The practical sessions were organised with the objectives of giving participants experience of a wide variety of automated instrumentation, and providing opportunities for discussing practical matters in depth, both with the representatives of the manufacturers and with independent experts, i.e. the course lecturers and tutors. With so many companies being represented it was impossible to give everybody 'hands on' experience of every instrument, but the combination of a formal allocation of experiments for some of the time and a free choice for the rest of the time satisfied most people. The manufacturers also, in the main, entered into the spirit of the school and this was much appreciated by the participants. The manufacturers also benefitted from having a captive audience and being able to discuss their instruments on a one to one basis in more calmer surroundings than an exhibition hall. They learnt a lot about their customers and

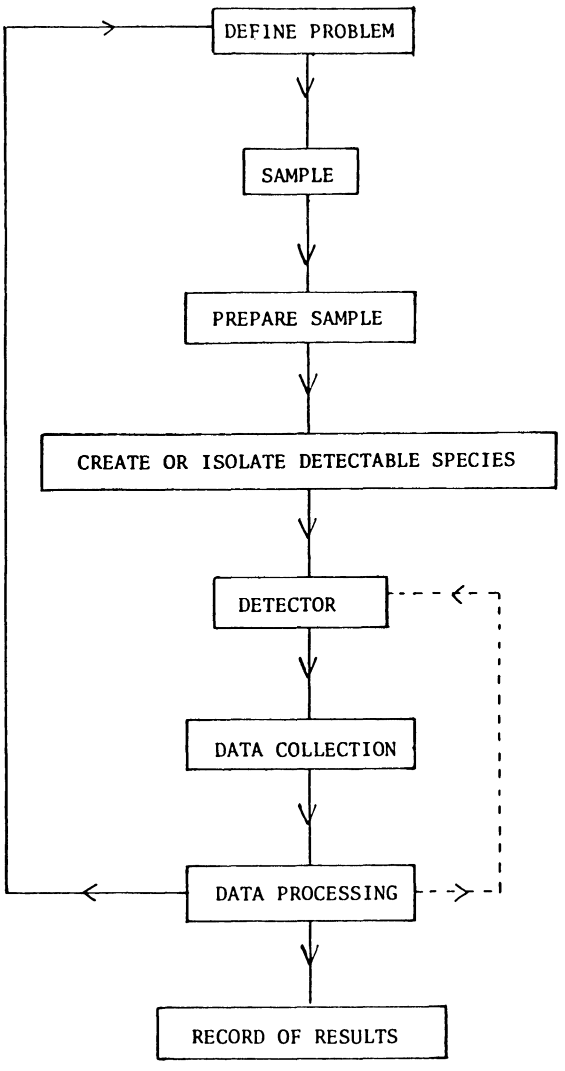

*Figure 1. A general scheme of automated analysis.*

their needs, and thus the school may be of long term benefit to all in automation.

**Student response**

Analysis of the course evaluation questionnaires completed by the students showed an extremely high level of approval. Thus it may be assumed that the objectives of the Summer School were met, but, like all good experiments, it raised many questions and provoked a number of reflections on the theme of 'education in automation'.

### Reflections on the educational significance of the Summer School

It has been argued elsewhere that successful developments in analytical chemistry take place as a consequence of dynamic interaction between theory, technique and analytical problems [1]. This led to the concept of the 'Analytical Trinity' and logo:

Technique ⇌ Theory
⇖⇘ ⇗⇙
Problem

It provides a convenient framework in which to discuss the achievements of the Summer School and the lessons to be learnt from it.

**Theory**

A uniform approach to the theory of automatic analysis was most notable by its absence. The elements of a complete theory were often discussed, and indeed a very useful diagram (Figure 1) summarising the various aspects of automation was presented, in several forms by a number of lecturers. However, most lectures were concerned with one particular aspect to the exclusion of others. A more generally accepted theory would help define and develop automatic analysis as a separate branch of analytical chemistry. This represents a challenge for the future.

One criterion for automatic analysis as defined by the IUPAC definition, is the presence of a feedback loop in the system. This seems a rather facile basis for characterisation. For most people the chief interest and distinguishing feature of automation is the capability of producing a large number of results with a high rate of sample throughput, usually for long periods. The motivation for setting up such an analytical service may be political, managerial or scientific, but, in all cases, the cost and organisation of the analysis are on a much greater scale than conventional manual analyses. In automated systems the amount of data generated also poses a number of implications. On the one hand, it must be processed and presented in a useable form, on the other, it can be used to help improve the analytical step (optimisation), to refine the initial statement of the problem and to maintain a continuous statistical check on the quality of the analysis. The reliability of results and the stability of apparatus is of greater significance with automatic analysis than with conventional. Hence, there is an additional analytical factor. Thus, those specialising in the design and development of automatic analysis instruments need to have a grounding in systems analysis, operational research, market research, network analysis, statistics, data processing, optimisation techniques and electronics in addition to the standard analytical techniques.

This may seem to over emphasise the problem but is is obvious that for chemists entering automation the novelties lie in management aspects and data processing.

It was often said during the school, that the definition of the problem was the most difficult part of the analysis, because of the economic and management problems associated with its solution. This was aptly illustrated by the course problem. Every tutorial group had great difficulty in deciding how, and on what basis, representative samples were to be taken and how the analytical results for each sample were to be recorded, collated and reported. It was a far cry from conventional analytical chemistry. Similarly, the exciting glimpses of what can now be achieved in the realms of data collection and evaluation showed what could be gained by applying advanced computational techniques to analysis. For example the introduction of Vidicon detectors enable sets of spectra to be taken at 10 m sec intervals over a period of 1 sec, this produces 100 spectra from which plots of absorbance as a function of time at various wavelengths can be reconstructed. Also Simplex optimisation methods can be used to set up the operating parameters of a complex apparatus to provide the maximum signal output.

All of the basic theory is currently available, from this the basic theory for automatic analysis must be synthesised. The result must be rigorous enough to underpin and effectively define automatic analysis, but it must be simple enough to be understood and readily accepted. Given the tendency for specialists in management and the mathematical skills to confuse straightforward ideas with jargon and equations, presentation of automation theory will not be easy. The lectures given during this summer school however proved that it can and should be done.

### Technique

There is no doubt that the Autoanalyser, Gemsaec, the Autolab and similar devices are of the utmost importance in analytical laboratories requiring a high sample throughput. There is equally, no doubt that they are far too expensive for undergraduate teaching laboratories.

To an academic, therefore, one of the most cheering features of the course was the demonstration of what could be done with DIY apparatus. For example a solvent extraction apparatus based on solenoid operated valves and developed in house by an automation development team was described. The construction of such an apparatus would be a worthwhile academic project, whilst its routine use would make basic, but tedious, experiments on solvent extraction possible.

The principles of continuous flow analysis are very well demonstrated by flow injection analysis, and this technique can also be introduced on a DIY basis.

The easily programmed microcomputer will also have a significant impact on the teaching of automated analysis. It is not difficult to envisage projects to develop automatic discrete analysers or to control continuous flow systems being based on interfacing minicomputers to readily available simple instruments.

Thus, there is considerable scope for the introduction of automated analysis into undergraduate teaching, and there are many advantages to be gained. Experience with flow injection analysis at the author's laboratory shows that the students find it interesting, that important chemical principles are illustrated simply and that it helps to have a high sample throughput. The speed of analysis results in a saving on instrumentation – one piece of FIA apparatus set up for a photometric determination can be used in one lab period by many more students than a spectrophotometer. It also means that the analytical results can be analysed statistically and that this important subject can be introduced to the students with the minimum of pain.

### Problems

On the course, one of the most valuable educational exercises was derived from the course problem. It brought home to the students the true complexity of modern analysis. The introductory statement of the objective is simple enough:

'As a member of the 'Transautomatia' Government Analyst's laboratory you have been asked to undertake work to determine the tar, nicotine and carbon monoxide levels produced by cigarettes available on the home market. This requires a carefully standardised and reproducible approach if the results are to be of value for comparative purposes. The Government wishes to publish twice yearly league tables similar in format to those produced by the UK government. These list the brands available in ascending order of tar level, grouping them in 1 mg increments, and should also show both nicotine and CO values. There are around 120-150 different cigarette brands currently available on the Transautomatia market'. The individuals were asked to develop a strategy for automating the analyses required.

However, it soon becomes apparent that the analyst has to be content with an arbitrary definition of tar, variability of the same brand of cigarette, the humidity, the variation of length of tobacco and filter and the need to establish a reproducible and reasonable puff profile.

A full account of one solution is given by Copeland and Stockwell [2]. Since the introduction of league tables of tar content, the pattern of smoking in the UK has changed significantly, cigarettes with low tar content being more favoured than before. This demonstrates rather well, the social and economic significance of analysis.

It was a challenging exercise and opinion was divided as to its worth, some thought it was a waste of time, others rated it the best part of the course. It has the merit of focussing the students' attention to a real world problem and to use the

### Conclusions

The lectures covered a wide range of topics and dealt with the more routine issues as well as novel techniques and instrumentation recently introduced. The tutorials and practical sessions enabled participants to discuss their own specific problems in depth and the course problem brought out many useful points and stimulated considerable discussion. Overall, the blend of theory, techniques and problems was such that most of the participants felt they had clearly benefitted. As an experiment in education by means of the short course it must be counted a success.

The stimulus provided by the interdisciplinary combination of management skills, science and technology was summarised for all, not just laboratory managers, by the concluding remarks of the opening lecture by J.K. Foreman:

'Perhaps the most significant problem the laboratory manager has concerns himself. To launch a laboratory into an automatic regime satisfactorily is a considerable achievement, involving, as has been summarised above, much learning and change of attitude. But he must stay abreast of development in his new arena. We have only just begun to extract, in any sort of cost-effective way, the maximum valuable information contained in a sample. Further developments in automation technology, if correctly oriented, are a powerful way of continuing this process and the laboratory manager's role in it can be a challenging and rewarding one'.

It may be that in the future this Summer School will be designated as the place where the concepts in education for automation began to gel.

REFERENCES

[1] Betteridge, D, Analytical Chemistry, 1976, 48, 1034A.
[2] Copeland, G.K.E. and P.B. Stockwell in "Topics of Automated Chemical Analysis". Volume 1. Eds. P.B. Stockwell and J.K. Foreman, 1979 Horwood, Chichester.

